# Human brain functional areas of unitary pooled activity discovered with fMRI

**DOI:** 10.1038/s41598-018-20778-3

**Published:** 2018-02-05

**Authors:** Jie Huang

**Affiliations:** Department of Radiology, Michigan State University, East Lansing, MI 48824 USA

## Abstract

We report the discovery of human brain functional areas of unitary pooled activity (FAUPAs) using fMRI. A FAUPA is defined as an area in which the temporal variation of the activity is the same across the entire area. This dynamically unitary activity implies a perfect temporal correlation everywhere within the FAUPA for the activity-induced BOLD response, i.e., the corresponding Pearson correlation coefficient (R) is 1 for the BOLD responses of any two locations within the FAUPA. A novel method was developed to identify the FAUPA. In this study, nine subjects had a resting-state (rs) fMRI and a task-fMRI. FAUPAs have been identified for both rs- and task-fMRI, and their determination was objective and automatic, with no *a-priori* knowledge. The group mean of R was 0.952 ± 0.004 for the rs-fMRI and 0.950 ± 0.002 for the task-fMRI, showing the dynamically unitary activity within each FAUPA. For the finger-tapping (FT) task, the group-mean BOLD signal time course of the FT-associated FAUPAs in the primary motor cortex was almost perfectly correlated with the FT-induced ideal response (R = 0.9330, P = 1.8 × 10^−56^), confirming the association of the FAUPA with the task. A task-associated FAUPA may play the role of a functional unit for a particular neural computation.

## Introduction

The non-invasive blood oxygenation level dependent (BOLD) functional magnetic resonance imaging (fMRI) technique has evolved as a major neuroimaging tool for studying functional organization of neural activity in the human brain at a large-scale^1–3^. To identify areas of the cortex activated by a task, the general linear model (GLM) is currently the most popular statistical approach^4,5^. It sets up an expected ideal response and fits it with the signal time course on a voxel-by-voxel basis so as to generate a map of t or z statistics. The activated voxels are then identified with significance levels exceeding a chosen threshold. The areas identified depend on the threshold chosen, and different thresholds yield different areas. Misidentified areas may occur if the significance level is not properly chosen, as evidenced in the clusterwise inference^6^. In addition, such a modelling approach requires a priori knowledge of the time course of the stimuli or tasks that are used to generate the ideal response. The approach is not suitable when the time course of the expected activity is unknown, such as when subjects perform tasks in a natural way without any restrictions. There are other approaches that make far fewer assumptions about the time course of the expected ideal response^5^. Most of these are based on some sort of decomposition of the data into time courses and spatial patterns that are uncorrelated (singular value decomposition, principal components analysis, or independent components analysis). Although these approaches are useful to identify areas that are uncorrelated, they may not be able to identify all areas in which activation occurs in response to a task, particularly those areas that are weakly correlated with each other. In this study, we present a novel method of identifying functional areas with no *a-priori* knowledge. The method avoids the short-comings identified above and the identification of functional areas is objective and automatic for each individual subject.

In a typical fMRI study with a spatial resolution of 3 × 3 × 3 mm^3^ and a temporal resolution of 2 sec, an activated voxel may contain over one million neurons and its corresponding BOLD signal change measures the activity-induced change from a pooled activity of these million neurons^3^. This signal change also reflects a temporally averaged effect of the pooled activity within the time window of the slice acquisition, and the sequential acquisition of every 2 sec over a period of time results in a BOLD signal time course that reflects the underlying dynamic pooled activity over the time period. Using BOLD signal time course to determine stimulus-induced cortical activated areas has proven to be as reliable and reproducible as human brain fMRI retinotopic mapping that utilizes the retinotopically organized visual areas^7–9^. For simplicity we consider the primary visual area V1. The one-to-one relationship between the visual field and the V1 area suggests that a visual stimulation from a small visual field should *only* induce a BOLD response in the representation area of the small visual field in V1, but not other areas within V1. If the temporal variation of the stimulation is the same across the small visual field, the temporal variation of the BOLD response should also be the same across the entire representation area, i.e., the pooled activity is a dynamically unitary activity, forming a functional area of unitary pooled activity (FAUPA). This FAUPA should be separated from its adjacent area because the latter is not activated by the stimulation. As another example, consider performing a finger-tapping (FT) task. Simultaneously tapping five fingers of the right-hand should induce a dynamic response in the well-confined five-finger representation area of the left primary motor cortex, and the temporal variation of this dynamic response should also be the same everywhere within this well-confined area, forming a FAUPA that is specifically associated with the FT task. In general, performing any functional task requires dynamically mediated activities of many brain areas, and some of these areas, if not all, could be well-confined areas. If the temporal variation of the pooled activity in a well-confined area is the same everywhere, this area forms a FAUPA that is associated with the task. Accordingly, we may expect the existence of some FAUPAs when performing any functional task. This study reports the identification of this conceptually conceived FAUPA in the human brain with fMRI.

## Theory

Let *n*(*r,t*) represent a pooled activity, where *r* is the spatial location and *t* time. The corresponding activity-induced BOLD response *S*(*r,t*) may be described by the convolution of *n*(*r,t*) with a hemodynamic response function *h*(*t*), i.e., $S(r,t)\,=\,{\int }_{0}^{t}n(r,\tau )\cdot h(t-\tau )\cdot d\tau$^10–12^. For a FAUPA, as the temporal variation of the pooled activity is the same everywhere, we can write $n(r,t)\,=\,f(r)\cdot g(t)$, where *g*(*t*) denotes the temporal variation and *f*(*r*) the spatial variation across the area. This equation implies that the pooled activity is spatially coherent and temporally synchronized. Accordingly, we have $S(r,t)\,=\,f(r)\cdot G(t)$, where $G(t)\,=\,{\int }_{0}^{t}g(\tau )\cdot h(t-\tau )\cdot d\tau$, a generic time function characterizing the temporal variation of the BOLD response. For a given two positions of *r*_1_ and *r*_2_, the corresponding two BOLD responses are ${S}_{1}({r}_{1},t)\,=\,f({r}_{1})\cdot G(t)$ and ${S}_{2}({r}_{2},t)\,=\,f({r}_{2})\cdot G(t)$, respectively. The temporal correlation between *S*_1_ and *S*_2_ is perfect, *i.e*., the Pearson correlation coefficient (*R*) is 1, because *S*_1_ and *S*_2_ have an identical temporal variation. This is also true for any two positions, showing a perfect temporal correlation of the BOLD response everywhere within the FAUPA. When the FAUPA is measured with fMRI, the voxel-wise signal time course is ${S}_{i}(t)\,=\,{C}_{i}\cdot G(t)\,+\,{\delta }_{i}(t)$, where *C*_*i*_ is the coefficient for the *i*^th^ voxel and ${\delta }_{i}(t)$ denotes the physiological and instrumental noises. In an ideal situation without any physiological and instrumental noises, i.e., ${\delta }_{i}(t)\,=\,0$, the temporal correlation of *S*_*i*_(*t*) is perfect among all these voxels. We define the FAUPA’s signal time course $\overline{S}(t)$ as the mean of *S*_*i*_(*t*), i.e., $\overline{S}(t)\,=\,\frac{1}{N}{\sum }_{i=1}^{N}{S}_{i}(t)$, where *N* denotes the total number of voxels contained in the FAUPA. We denote the correlation coefficient of $\overline{S}(t)$ with ${S}_{i}(t)$ as *R*_*i*_, and define the FAUPA’s $\overline{R}$ as the mean of *R*_*i*_. When ${\delta }_{i}(t)\,=\,0$, we have $\overline{S}(t)\,=\,\overline{C}\cdot G(t)$, where $\overline{C}$ is the mean of *C*_*i*_. This results in *R*_*i*_=1 for every voxel, and consequently, $\overline{R}\,=\,1$, reflecting the dynamically unitary pooled activity. In reality, ${\delta }_{i}(t)\,\ne \,0$, the mean (μ) and standard deviation (σ) of *R*_*i*_ measure the overall strength of the correlation and its corresponding variation among the voxels, respectively. These μ and σ can be used to establish a statistical model to determine the FAUPA, and we have developed and tested a computational algorithm to identify FAUPAs^13^.

## Results

### FAUPAs identified with rs-fMRI

**Figure 1:**
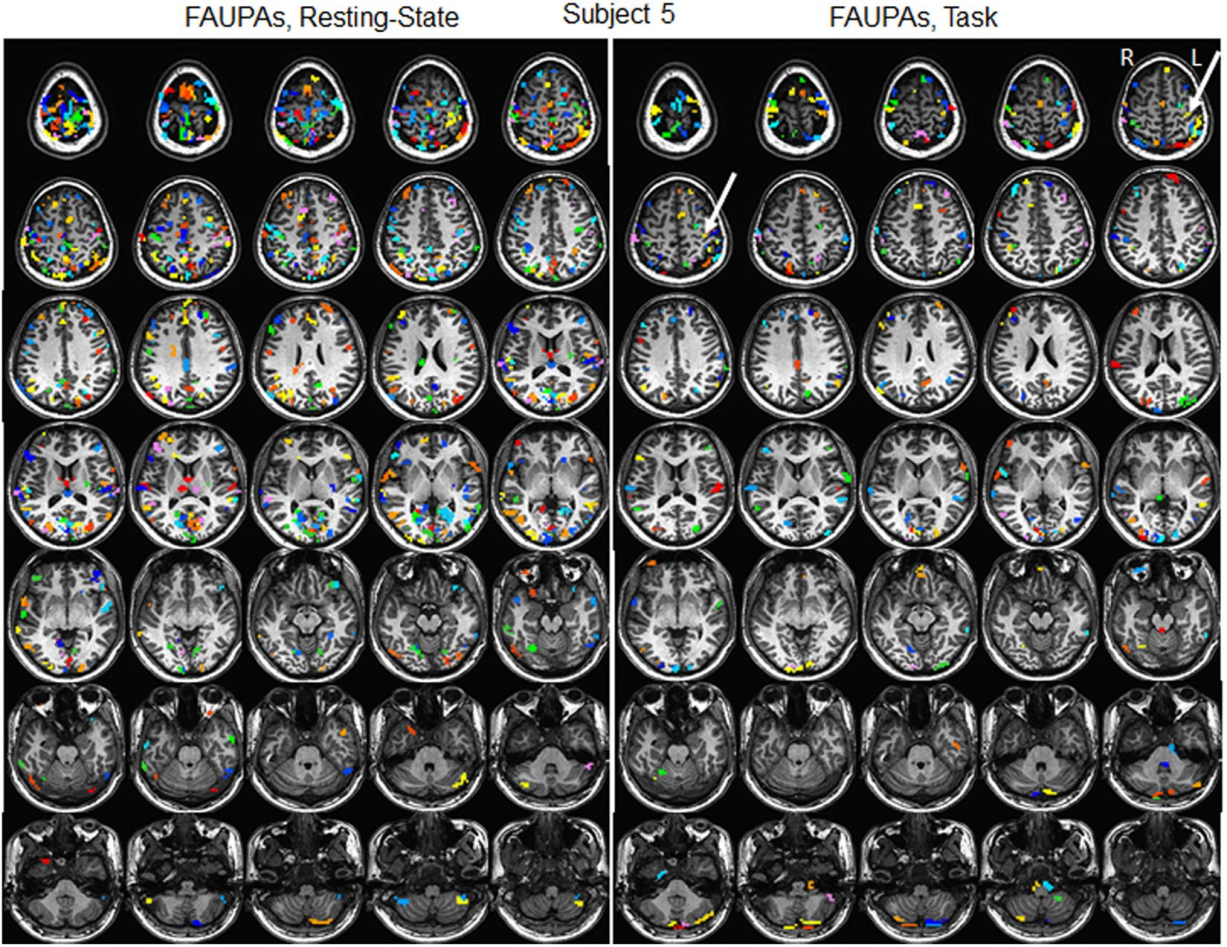
Illustration of FAUPAs for subject 5 (Table 1). Each colored cluster with all connected voxels of the same color represents a FAUPA, and a total of 16 colors are used to differentiate these FAUPAs. Resting State: a total of 433 FAUPAs; Task: a total of 203 FAUPAs. The arrow-pointed yellow-colored cluster on the left primary motor cortex illustrates a FT-associated FAUPA, i.e, its signal change time course appears to be associated with the FT task (Fig. 3). L: left; R: right.

**Figure 2:**
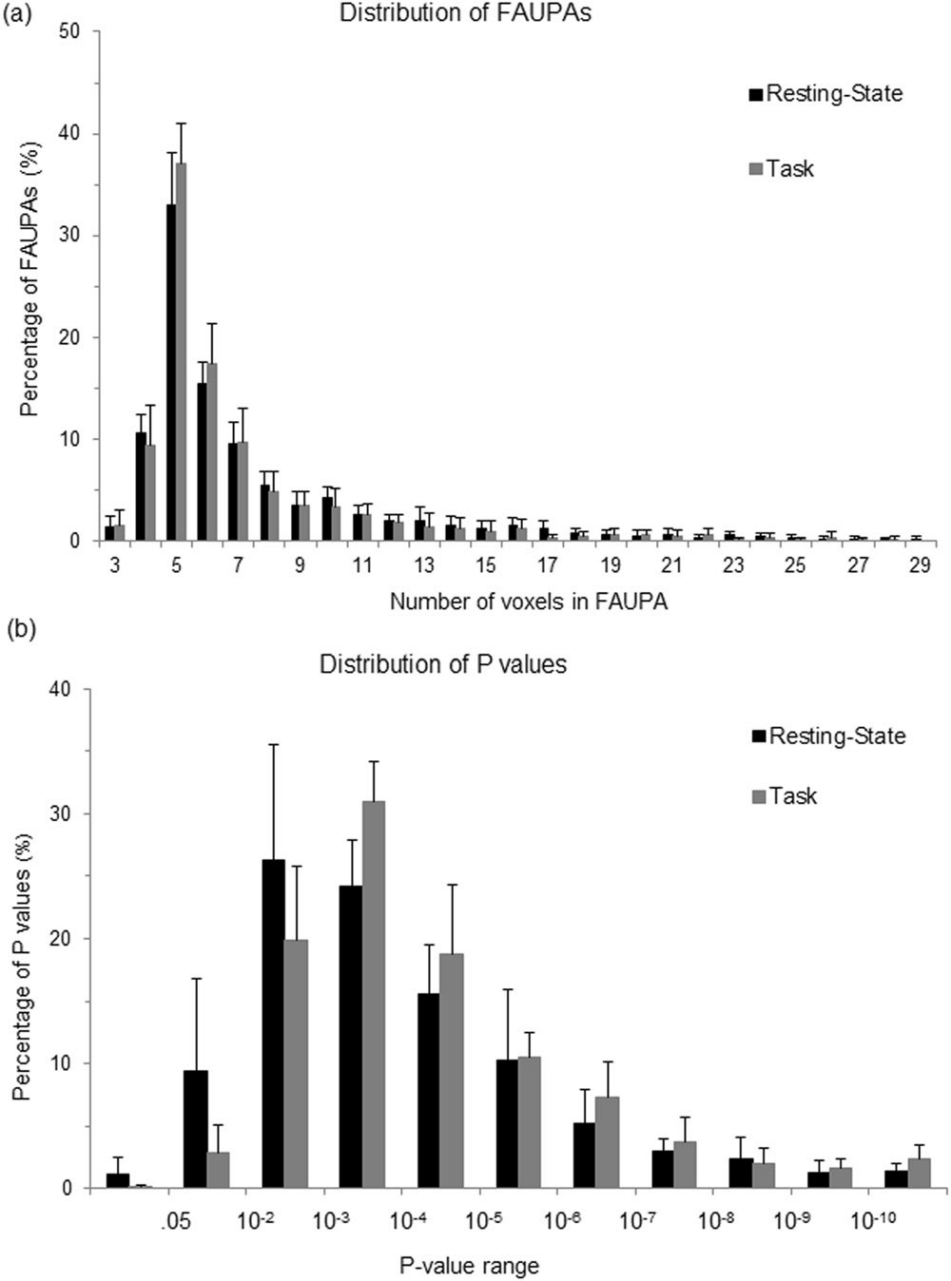
Comparison of histograms for both rs-fMRI and task-fMRI. (**a**) The histogram of group-mean FAUPAs as a function of the total number of voxels contained in a FAUPA; (**b**) The histogram of group-mean P values as a function of P-value range. The error bars indicate the corresponding standard deviations.

FAUPAs were detected for each of the nine rs-fMRI scans, and the total number of the identified FAUPAs across the whole brain varied from 121 to 751 with μ ± σ = 352 ± 207 (Table 1). The left panel in Fig. 1 illustrates the 433 FAUPAs for subject 5. The total number of voxels that a FAUPA contained varied from 3 to 29, and Fig. 2a plots their group-mean distribution. For each subject, the maximum, minimum, μ and σ of the $\overline{R}$ values are tabulated in Table 1. The range of the mean $\overline{R}$ values is from 0.945 to 0.957 for the nine subjects, and the group μ and σ of these mean $\overline{R}$ values are 0.952 ± 0.004, showing an almost perfect temporal correlation within each FAUPA.

**Table 1:** FAUPAs determined with rs-fMRI and task fMRI. Subj: subject; #: number; Max: maximum; and Min: minimum.

Subj	Resting-State					Task				
	#of FAUPAs	Pearson Correlation Coefficient R				#of FAUPAs	Pearson Correlation Coefficient R			
		Max	Min	μ	σ		Max	Min	μ	σ
1	224	0.991	0.889	0.956	0.016	58	0.981	0.862	0.947	0.019
2	385	0.992	0.874	0.957	0.018	139	0.991	0.901	0.949	0.019
3	537	0.992	0.863	0.951	0.017	153	0.985	0.899	0.950	0.015
4	209	0.995	0.821	0.952	0.019	69	1	0.905	0.951	0.018
5	433	0.990	0.866	0.953	0.018	203	0.983	0.901	0.950	0.015
6	383	0.993	0.839	0.953	0.017	286	1	0.856	0.953	0.018
7	121	0.977	0.818	0.945	0.022	155	1	0.878	0.950	0.018
8	128	0.986	0.850	0.948	0.018	114	0.978	0.887	0.948	0.015
9	751	0.995	0.886	0.957	0.018	280	1	0.879	0.950	0.018
μ	352	0.990	0.856	0.952	0.018	162	0.991	0.885	0.950	0.017
σ	207	0.006	0.026	0.004	0.002	82	0.009	0.018	0.002	0.002

### FAUPAs identified with task-fMRI

Similar as the rs-fMRI, FAUPAs were also detected for each of the nine task-fMRI scans. The total number of the identified FAUPAs across the whole brain varied from 58 to 286 with μ ± σ = 162 ± 82 (Table 1), significantly smaller than that of the rs-fMRI (one-tail paired t-test, P = 0.0041), showing the dependence of FAUPA on the functional state of brain. When compared with the rs-fMRI-identified FAUPAs, the right panel in Fig. 1 illustrates the 203 task-fMRI-identified FAUPAs for the same subject. The group-mean distribution of these task-fMRI-identified FAUPAs, however, is similar as that of the rs-fMRI-identified FAUPAs (Fig. 2a). For each subject, the maximum, minimum, μ and σ of the $\overline{R}$ values for these task-fMRI-identified FAUPAs are also tabulated in Table 1. The range of the mean $\overline{R}$ values is from 0.947 to 0.953 with a group μ ± σ = 0.950 ± 0.002, and is almost identical to that of the rs-fMRI-identified FAUPAs, further demonstrating the almost perfect temporal correlation within each FAUPA that is independent of the functional state of brain.

### Testing the separation of FAUPAs from their adjacent areas

To test the separation of a FAUPA from its adjacent area, a layer of one voxel width bordering the FAUPA was first generated. This resulted in a representative adjacent area. To examine whether the signal time course in this adjacent area is significantly different from the FAUPA’s $\overline{S}(t)$, we first computed the temporal correlation of a voxel’s signal time course ${S}_{i}(t)$ with $\overline{S}(t)$ for each voxel, and then compared the *R*_*i*_ values of the adjacent area with those of the FAUPA (one-tail t-test). The corresponding P value was then used to determine the separation of the FAUPA from its adjacent area; the FAUPA was separated significantly from its adjacent area if P < 0.05 (multiple comparisons uncorrected). Fig. 2b shows the histograms of the group-mean P values for both rs- and task-fMRI-identified FAUPAs. For the task-fMRI-identified FAUPAs, less than 0.1% of them having P values ≥ 0.05, showing that more than 99.9% of them were separated significantly from their adjacent areas. Similar results were obtained for the rs-fMRI-identified FAUPAs; less than 1.2% of the P values ≥ 0.05. Accordingly, more than 98.8% of these rs-fMRI-identified FAUPAs were separated significantly from their adjacent areas. Overall, more than 98.8% of the identified FAUPAs were separated significantly from their adjacent areas for both the resting and task states.

### Finger-tapping-associated FAUPA in the motor cortex

**Figure 3:**
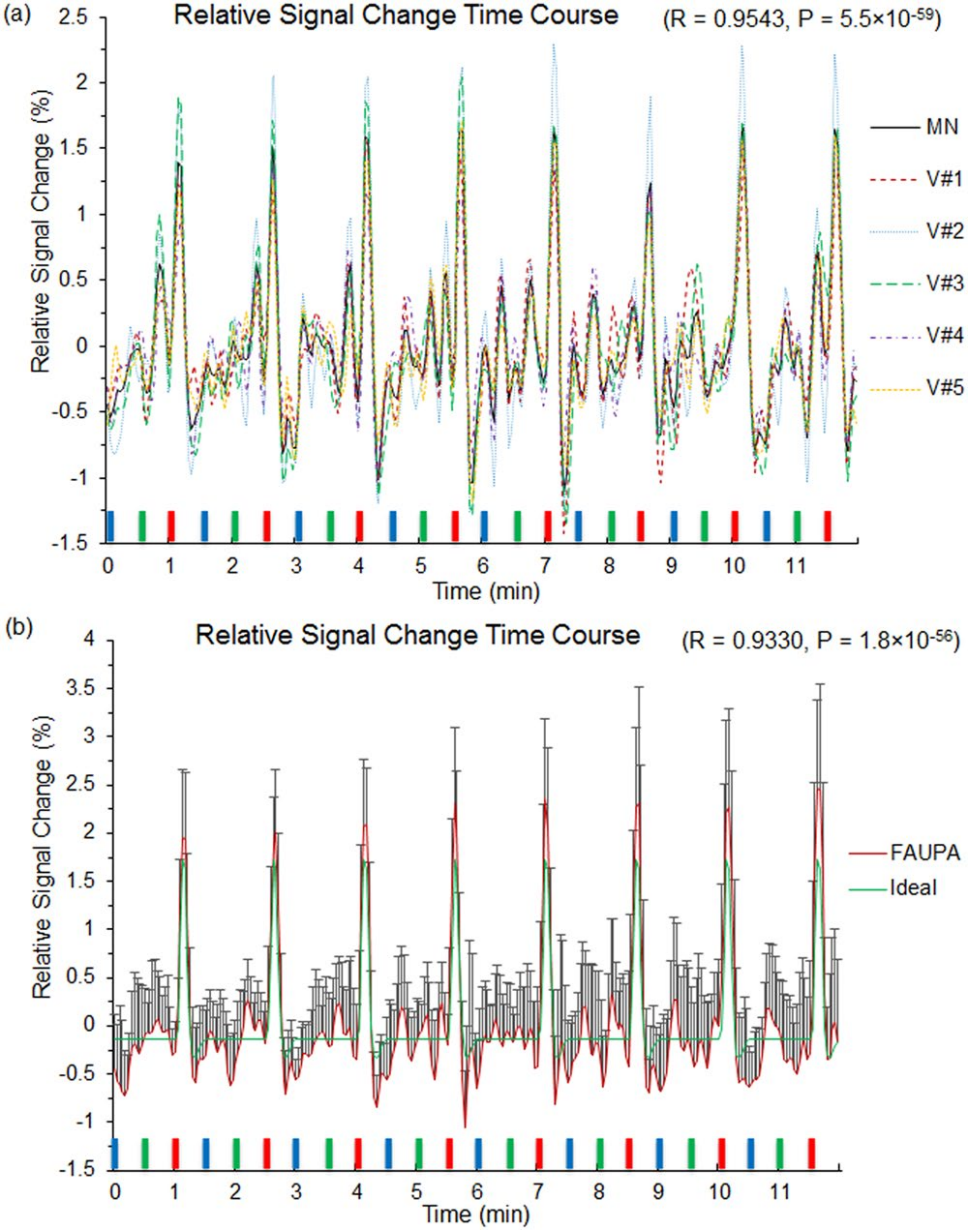
Signal time courses of FT-associated FAUPAs in the left primary motor cortex. (**a**) five signal time courses of the 5 voxels contained in the FT-associated FAUPA for subject 5 (Fig. 1), and the black solid line represents their mean (MN) signal time course. The blue, green and red bars represent the onset and duration of the three tasks of reading words, viewing patterns and tapping right-hand five fingers, respectively. A task-induced large signal change that is associated with the FT task is present for each of the eight tasks. V: voxel; (**b**) comparison of the ideal response signal time course with the group-mean signal time course of the nine FT-associated FAUPAs from the nine subjects. The error bar indicates the standard deviation.

We define a task-associated FAUPA as a FAUPA that is activated when the task is performed. To examine the association of FAUPA with task, we first identified a FAUPA in the left primary motor cortex with a signal time course that appears to be associated with the FT task, assuming that the temporal variation of this signal time course was induced by the FT task alone. Such a putative FT-associated FAUPA was identified for each subject, and was located in the similar area of the left primary motor cortex across all nine subjects. The arrow-pointed yellow-colored cluster in Fig. 1 illustrates the identified FT-associated FAUPA for subject 5, and this FAUPA contains a total of 5 voxels across slices 5 and 6. The plot in Fig. 3a illustrates the relative signal change time courses for these 5 voxels. The solid black line in this plot shows the FAUPA’s signal time course $\overline{S}(t)$, and all the five voxels’ signal time courses are almost perfectly correlated with the FAUPA’s signal time course, reflected in the FAUPA’s mean *R* value μ ± σ = 0.954 ± 0.013 and showing the unitary pooled activity of the FAUPA. A task-induced large signal change is present for each of the eight FT trials. This is also true for each identified FT-associated FAUPA for the other eight subjects.

To test the association of these nine FAUPAs with the FT task, we generated an ideal response signal time course based on the temporal paradigm of the FT task alone, using the 3dDeconvolve program in AFNI with the convolution kernel SPMG3 (http://afni.nimh.nih.gov/afni). (This signal time course was scaled by a factor of 2.) We then computed its relative signal change time course by subtracting the signal time course with its mean and dividing that change with the mean. Then, the temporal correlation of this ideal response with FAUPA’s relative signal change time course was computed for each of the nine FAUPAs. The *R* value was ranged from 0.5781 (P = 1.0 × 10^−22^) to 0.8033 (P = 2.6 × 10^−42^) with μ ± σ = 0.7025 ± 0.0792 (*R* = 0.7025, P = 9.1 × 10^−33^). The group-mean relative signal change time course is almost perfectly correlated with the ideal response, showing a statistically highly significant association of the FAUPAs with the FT task (*R* = 0.9330, P = 5.3 × 10^−56^) (Fig. 3b).

### The effects of spatial filtering on the detection of FAUPAs

**Figure 4:**
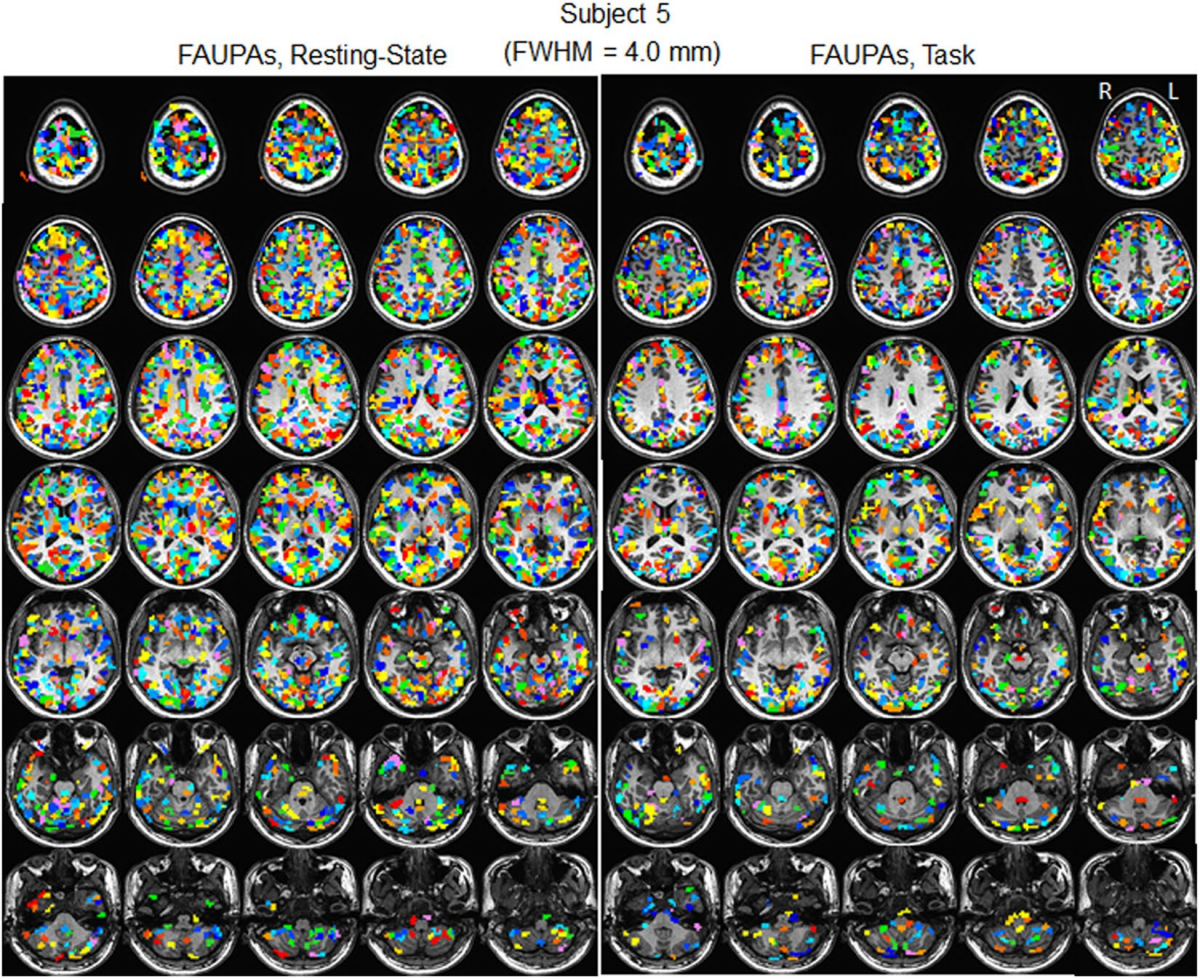
Illustration of the effect of spatial blurring with FWHM of 4.0 mm on the detected FAUPAs for subject 5. Each colored cluster with all connected voxels of the same color represents a FAUPA, and a total of 16 colors are used to differentiate these FAUPAs. Resting State: a total of 2192 FAUPAs; Task: a total of 1383 FAUPAs. L: left; R: right.

**Figure 5:**
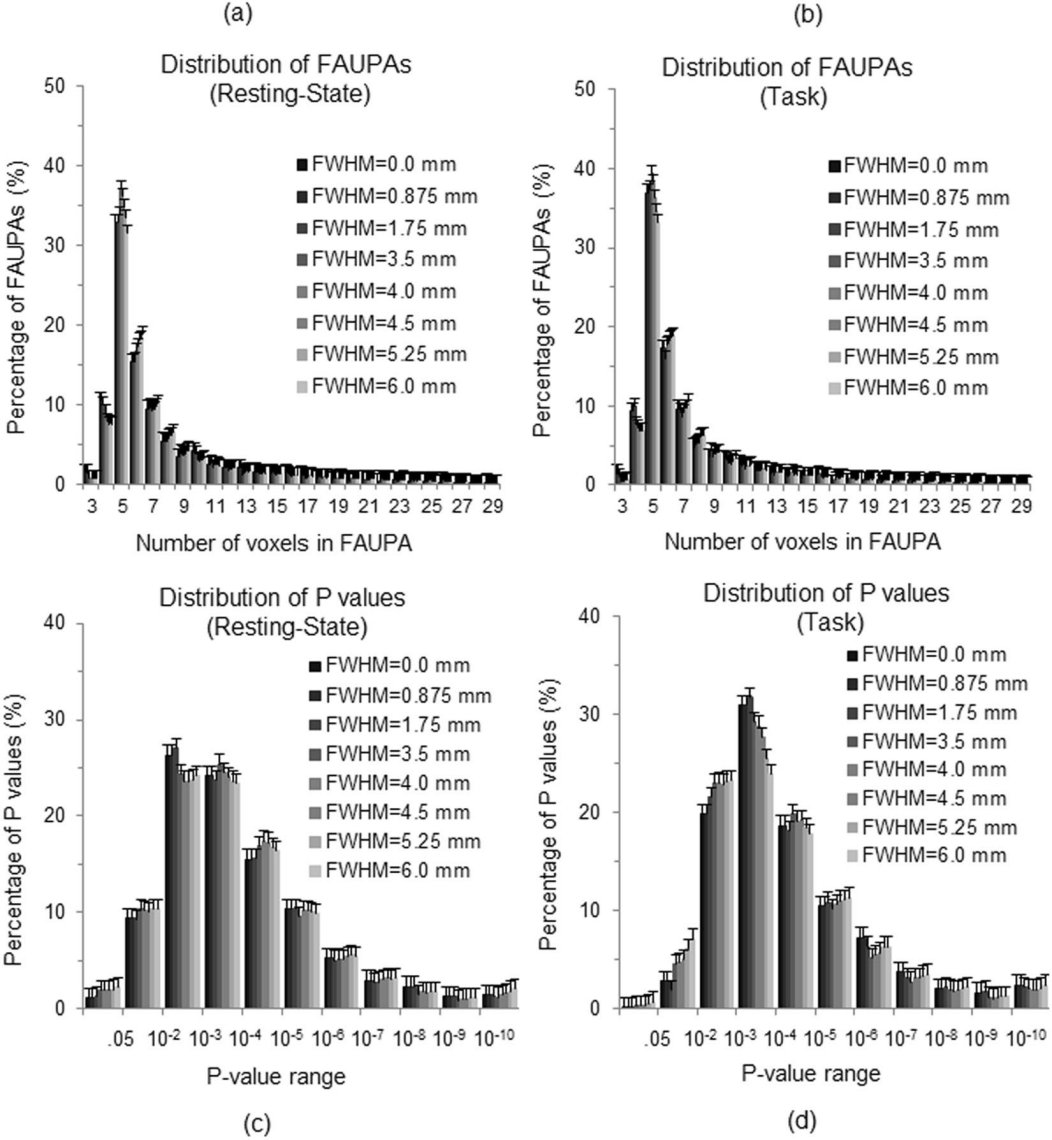
Comparison of the effects of spatial blurring on the histograms for both rs-fMRI and task-fMRI. (**a**) and (**b**): The effects of FWHM on the histogram of group-mean FAUPAs as a function of the total number of voxels contained in a FAUPA; (**c**) and (**d**): The effects of FWHM on the histogram of group-mean P values as a function of P-value range. The error bars indicate the corresponding standard deviations.

We investigated the effects of spatial filtering (blurring) on the detection of FAUPAs with seven FWHM value of 0.875, 1.75, 3.5, 4.0, 4.5, 5.25 and 6.0 mm. Table 2 tabulates the total number of FAUPAs detected as a function of FWHM. For each subject, except the FWHM of 0.875 mm which produced the same results as that without the spatial blurring, the blurring increased the total number of FAUPAs with increasing FWHM, regardless of the brain state, and all these increases were statistically significant (one-tail paired-ttest, max P = 2.1 × 10^−4^). When compared with that without the blurring, the FWHM of 4.0 mm increased the total number of FAUPAs by an average of 5.2 times for the resting-state and of 8.1 times for the task, respectively. Fig. 4 illustrates these increased FAUPAs for subject 5. Although the spatial blurring significantly increased the total number of FAUPAs, the histogram of these FAUPAs remained similar as that without the blurring for both resting-state and task (Fig. 5a). The test of separation of these FAUPAs from their adjacent areas also showed a similar histogram of P values for all FWHM values, regardless of the brain state (Fig. 5b).

**Table 2:** The effects of the spatial smoothing on the detection of FAUPAs. Subj: subject. FWHM: full-width-half-maximum.

	Subj	FWHM (mm)							
		0	0.875	1.75	3.5	4.0	4.5	5.25	6.0
Resting-State	1	224	224	254	989	1464	1945	2790	3433
	2	385	385	395	1314	1823	2341	3025	3730
	3	537	537	589	1661	2114	2622	3283	3891
	4	209	209	231	1028	1418	1820	2461	3121
	5	433	433	458	1640	2192	2752	3422	3876
	6	383	383	425	1395	1773	2208	2818	3319
	7	121	121	144	872	1373	1968	2756	3360
	8	128	128	151	891	1401	2045	2897	3612
	9	751	751	814	2242	2804	3247	3854	4205
	μ	352.3	352.3	384.6	1336.9	1818	2327.6	3034	3616.3
	σ	206.9	206.9	219.8	454.3	480.0	466.3	420.7	341.9
Task	1	58	58	68	522	924	1442	2285	3112
	2	139	139	163	718	1123	1592	2388	3167
	3	153	153	172	758	1113	1618	2392	3114
	4	69	69	72	549	903	1328	2074	2770
	5	203	203	231	982	1383	1913	2559	3146
	6	286	286	306	1170	1644	2044	2707	3230
	7	155	155	184	1075	1611	2217	2956	3484
	8	114	114	127	776	1292	1801	2645	3446
	9	280	280	301	1285	1784	2369	3143	3812
	μ	161.9	161.9	180.4	870.6	1308.6	1813.8	2572.1	3253.4
	σ	81.7	81.7	87.0	270.5	320.3	352.8	334.3	294.7

**Figure 6:**
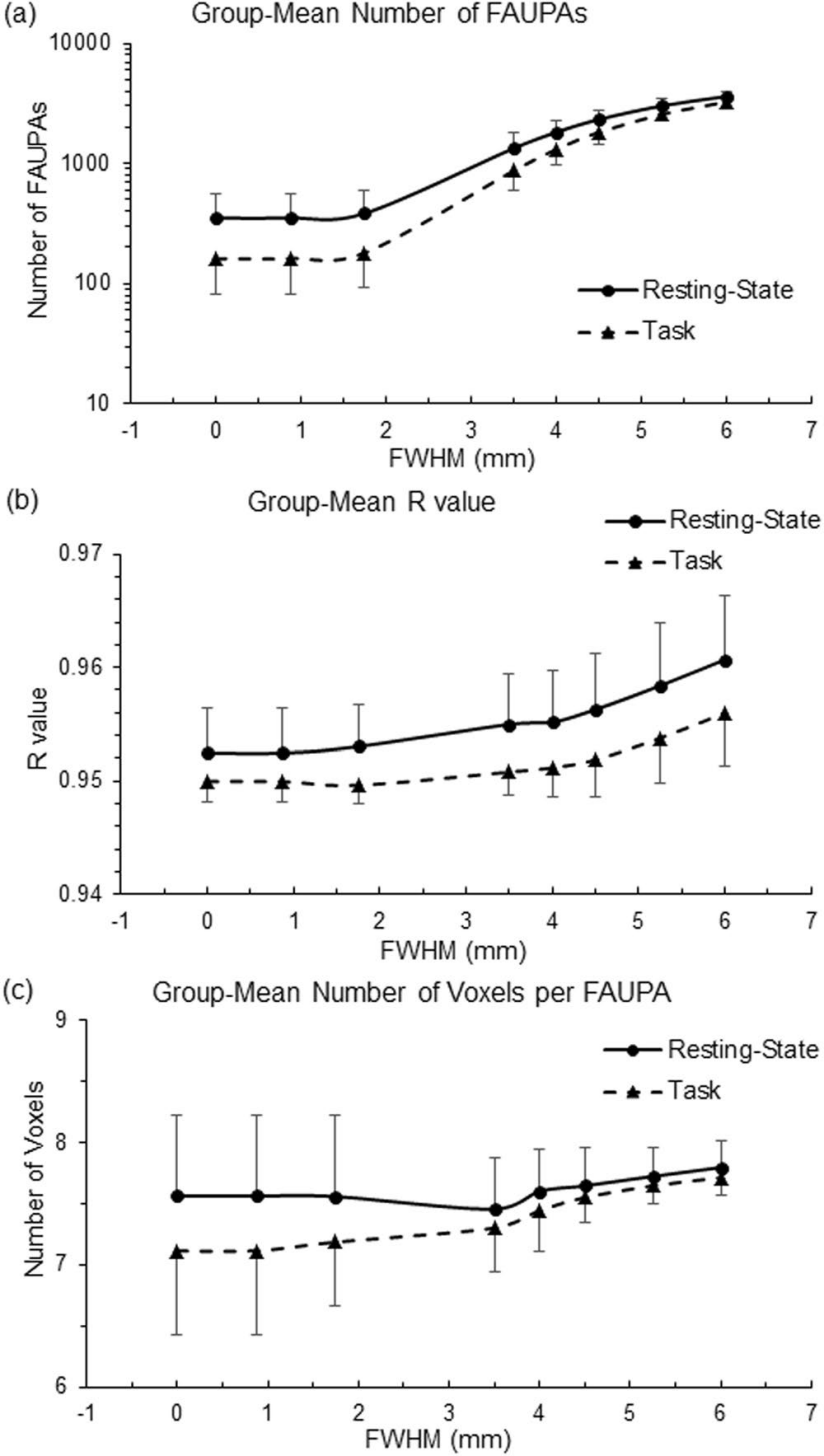
Illustration of the effects of spatial filtering on the detected FAUPAs. (**a**) The group-mean total number of FAUPAs as a function of FWHM; (**b**) The group-mean *R* values as a function of FWHM; and (**c**) The group-mean number of voxels per FAUPA as a function of FWHM. The error bars indicate the corresponding standard deviations.

**Figure 7:**
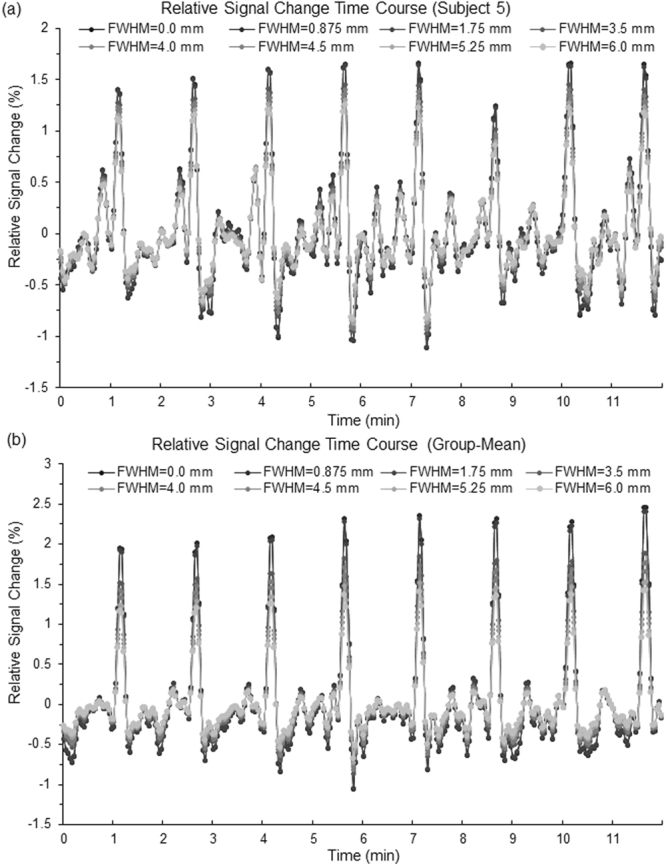
Illustration of the effect of spatial filtering on the signal time courses of the FT-associated FAUPAs identified in the left primary motor cortex. (**a**) The variation of the relative signal change time course with FWHM for subject 5; and (**b**) The variation of the group-mean relative signal change time course with FWHM.

To further investigate the effects of spatial blurring on the detected FAUPAs, we computed the group-mean values of (1) the total number of FAUPAs, (2) their corresponding FAUPA-mean *R* values, and (3) their corresponding mean number of voxels per FAUPA (Fig. 6). The increase of the group-mean of the total number of FAUPAs with increasing FWHM was approximately exponential (Fig. 6a), and the FWHM of 6.0 mm increased the total number of FAUPAs by an average of 10.3 times for the resting-state and of 20.1 times for the task, respectively. For each subject, the FAUPA-mean of *R* values was computed over all FAUPAs for each FWHM. Then, the group-mean of the FAUPA-mean *R* values was computed for each FWHM, and this group-mean *R* values increased slightly with increasing FWHM as shown in Fig. 6b. Similarly, for each subject, the mean number of voxels per FAUPA was computed over all FAUPAs for each FWHM. Then, the group-mean of the mean number of voxels per FAUPA was computed for each FWHM, and this group-mean number of voxels per FAUPA varied slightly with FWHM as demonstrated in Fig. 6c. Fig. 7 illustrates the effect of spatial blurring on the signal time courses of the FT-associated FAUPAs identified in the left primary motor cortex. As can be seen, the spatial blurring reduced not only the noise but also the signal, and the larger the FWHM, the bigger the reduction. The overall pattern of these signal time courses, however, remained unchanged.

### Discussion and Conclusions

The conceptually conceived brain dynamic neural activity-associated FAUPA was experimentally verified in this study. The identified FAUPA has these characteristics:

1. It is a dynamic neural activity-associated functional area that is separated from its adjacent area;
2. The underlying pooled activity is a dynamically unitary activity, i.e., the activity is spatially coherent and temporally synchronized across the entire area;
3. This unitary activity is characterized by a generic time function *G*(*t*) that relates to the FAUPA’s $\overline{S}(t)$ via $\overline{S}(t)\,=\,\overline{C}\cdot G(t)\,+\,\bar{\delta }(t)$;
4. FAUPA is brain-state dependent;
5. Different FAUPAs can be formed in the same cortical area for different brain states;
6. The mean size of FAUPA is about 300 mm^3^.

The determination of FAUPA is objective and automatic with no requirement of a priori knowledge of the activity-induced ideal response signal time course. This determination is based on the assumption: the temporal variation of the underlying pooled activity is the same across the entire area, i.e., the pooled activity is a dynamically unitary activity. This assumption implies a perfect temporal correlation everywhere, i.e., $\overline{R}$ = 1. The assumption was verified by the observed $\overline{R}$ values of the identified FAUPAs. For the resting state, the range of the mean $\overline{R}$ values is from 0.945 to 0.957 with a group μ ± σ = 0.952 ± 0.004 for the nine subjects (Table 1). For the task state, the range is from 0.947 to 0.953 with a group μ ± σ = 0.950 ± 0.002, similar as that of the resting state. This almost perfect temporal correlation across the entire area shows that the underlying pooled activity is indeed a dynamically unitary activity, and the FAUPA’s signal time course measures this pooled activity.

The determination of FAUPA is self-consistent, i.e., for each identified FAUPA, the statistical model used to determine the FAUPA is satisfied. There are FAUPAs adjacent to each other as shown in Fig. 1. Two adjacent FAUPAs may show a very similar behaviour, i.e., their time courses are very similar to each other, and they may or may not perform the same local neural computation. If they do not perform the same local neural computation, then they are correctly identified. If they do perform the same local neural computation, then they should be one FAUPA combining the two together, and this means that the presented statistical model is not sensitive enough to correctly identify this FAUPA. How to test them is beyond the scope of this paper. It’s worth mentioning that the maximum number of voxels contained in a detected FAUPA was 29 because of the chosen cutoff voxel number of 29. (This threshold was chosen to improve the efficiency of computation). FAUPAs containing more than 29 voxels may exist, but the chosen threshold limited the detection of these FAUPAs in this study. A further test may determine whether this threshold could be removed from the detection. In addition, the presented statistical model chose 4 voxels as an initial seed to detect FAUPA, and a test with 5 voxels as an initial seed produced similar results for the detected FAUPAs. However, the reliability of the detection with different initial seeds requires further investigation.

The separation of a FAUPA from its adjacent area was verified by testing the temporal correlation of the signal time courses between the FAUPA and its adjacent area. More than 98.8% of the identified FAUPAs show a significant difference from their adjacent areas for both the resting state and the task state (Fig. 2b). Although the majority of the FAUPAs show statistically significant separations from their adjacent areas, there are about 1% of them that do not significantly separate from their adjacent areas. Even for those that do show a significant separation, there are still a few adjacent voxels that may have signal time courses similar as that of the FAUPAs because the determination of FAUPA used a statistical model. How to improve the accuracy of this determination requires further investigation.

Spatial blurring is routinely used in the pre-processing of fMRI images to prepare the images for statistical analysis, and its one main purpose is to reduce the noise level whilst retaining the underlying signal^14^. Spatial blurring is effectively a local averaging function. It leads to a partial cancellation of the random noises from the adjacent voxels and consequently results in a reduced noise level. In this study, the isotropic voxel had a size of 3.5 mm. For the FWHM of 0.875 which is only 25% of the voxel size, we should expect that the corresponding blurring has no effect on the detection of FAUPAs, and this is confirmed by the same results compared with that without blurring. The spatial blurring with all other FWHM values effectively reduced the noise level and resulted in a significantly increased total number of FAUPAs; the larger the FWHM, the bigger the total number of FAUPAs (Fig. 6a). (The spatial blurring-reduced noise is also reflected in the slightly increased *R* value with increasing FWHM as shown in Fig. 6b.) The new FAUPAs revealed with the spatial blurring have the similar characteristics as those identified without blurring (Figs 5, 6b,c). A noticed difference between FAUPAs identified without blurring and with blurring is that the spatial blurring also reduced the signal as shown in Fig. 7; the larger the FWHM, the bigger the reduction. This reduction effect to the signal should be expected. For a given voxel, as the voxel has 26 adjacent voxels and most of these adjacent voxels may not have a signal similar as the voxel’s signal, a spatial blurring of these voxels should result in a reduced signal for the voxel, consistent with the observation. The majority of the FAUPAs were located within the cerebral cortex, though a few of them were outside of the cortex, regardless of the FWHM value and the brain state (Figs 1 and 4). For those within the cerebral cortex, they were located mainly within the grey matter, indicating an association of their dynamic signal changes with the underlying neural activities. Considering the approximately exponential increase in the total number of FAUPAs with increasing FWHM (Fig. 6a), an appropriately chosen FWHM may yield FAUPAs that form a reliable brain-state dependent functional parcellation of the grey matter, but its verification is beyond the scope of this paper.

The total number of voxels contained in a FAUPA varied from 3 to 29, and there were about 35% of the FAUPAs containing five voxels each, followed by ~16% containing six voxels, etc. (Fig. 2a). There were about 75% FAUPAs with voxels from 4 to 8. The group-mean number of voxels per FAUPA was 7.56 ± 0.65 for the rs-fMRI and 7.11 ± 0.68 for the task-fMRI, respectively, and these numbers remained similar for all FWHM values (Fig. 6c). In this study, the isotropic voxel had a size of 3.5 mm. Accordingly, the mean size of the FAUPAs was 324 ± 28 mm^3^ for the rs-fMRI and 305 ± 29 mm^3^ for the task-fMRI, respectively. This leads to the conclusion: the mean size of FAUPA is about 300 mm^3^.

The association of FAUPA with task was verified by testing the temporal correlation of the FT-associated FAUPA’s signal time course with the FT-induced ideal response signal time course at both the individual level and the group level (Fig. 3). At the individual level, each of the eight FT tasks induced a large signal change that was significantly correlated with the task for each individual subject (the minimum *R* = 0.5781, the corresponding maximum P = 1.0 × 10^−22^). At the group level, the group-mean signal time course was almost perfectly correlated with the FT-induced ideal response signal time course (*R* = 0.9330, P = 5.3 × 10^−56^), showing the association of these FAUPAs with the FT task (Fig. 3b). As the pooled activity within a FAUPA is spatially coherent and temporally synchronized, a task-associated FAUPA may play the role of a functional unit for a particular neural computation.

A neural network may be characterized as a set of nodes and edges that represent system elements and their interrelations, and the communication between networks is through hubs that connect these networks^15,16^. Accordingly, a task-specific network may be characterized by a set of nodes and hubs that constitute the system, and when the task is performed, the task performance activates all these nodes and hubs. The edges of the network may be characterized by the functional connectivity among the nodes and hubs. There are three tasks of word-reading (WR), pattern-viewing (PV) and FT in this study, and therefore there are total of seven combinations among these three tasks: (1) FAUPAs associated with the WR task alone; (2) those associated with the PV task alone; (3) those with the FT task alone; (4) those with both WR and PV tasks; (5) those with both WR and FT tasks; (6) those with both PV and FT tasks; and (7) those with all the three tasks. Task-associated FAUPAs in each category were identified for each subject, and three task-specific networks were identified for the three tasks (data not presented). For example, the FT network was comprised of all task-associated FAUPAs in the four categories of (3), (5), (6) and (7). Each FAUPA in category (3) constituted a node for the FT network because the FAUPA was activated only when the FT task was performed, but not the other two WR and PV tasks. Each FAUPA in category (5) constituted a hub that connected the FT network with the WR network because the hub was activated when either the FT or WR task was performed, and, similarly, each one in category (6) constituted a hub that connected the FT network with the PV network. Each FAUPA in category (7), however, constituted a hub that connected all the three networks because the hub was activated by all three tasks. The dynamic activity of a task-specific network may be characterized by the temporal changes of both activation and functional connectivity of the network, i.e., the activation change of each FAUPA and the functional connectivity change of any paired FAUPAs within the network from trial to trial. Accordingly, analysing the signal time courses of all FAUPAs within the network may reveal the dynamic network activity from trial to trial. This dynamic network activity from trial to trial may offer a means of systematically manipulating task performance and measuring corresponding network activity change to test the causal relationship between network activity and human task performance. We have completed such an analysis, and will report the results in another paper.

The importance of rs-fMRI technique is highlighted by the $40 million NIH-funded Human Connectome Project. It is a standard method to use correlations in the spontaneous temporal fluctuations in the signal time courses to deduce functional connectivity among different cortical areas and model functional networks^17–19^. The technique provides an indirect, but invaluable indicator of grey-matter regions that interact strongly and in many cases are connected anatomically^20^. It allows one to study functional connectivity in the brain by acquiring fMRI data while subjects lie inactive in the MRI scanner, and taking advantage of the fact that functionally related brain regions spontaneously co-activate. This technique is particularly suitable for clinical applications because the approach does not require the patient to perform a task and scans can be obtained in a relatively short amount of time. It is emerging as a mainstream approach for imaging-based biomarker identification, detecting variations in the functional connectome that can be attributed to clinical variables (e.g., diagnostic status)^21^. Regarding rs-fMRI analysis, the team responsible for the Human Connectome Project stated: “Emerging from the background of general connectivity estimation techniques such as seed-based correlation and independent component analysis, ‘connectome’ mapping often includes two stages: first the identification of a set of ‘nodes’ (through a parcellation of the brain’s grey matter), and secondly, estimation of the set of connections or ‘edges’ between these nodes, based on the fMRI time series associated with the nodes”^22^. They also stated: “Mapping the connectome is often assumed to begin with the parcellation of grey matter into (often non-overlapping) regions, … ***Ideally***, the regions are functionally specialised parcels, within each of which connectivities are relatively homogeneous — all locations within a parcel are assumed to have a similar general pattern of connectivity to locations in the brain outside the parcel”^22^. FAUPAs possess these characteristics and may serve as the described ideal parcels for mapping the human brain functional connectome. In addition, as both FAUPAs and FAUPA-associated networks are objectively identified for each individual, it may provide a means of objectively comparing functional networks across individuals that is essential for a possible application of the technique to clinical practice for diagnosing neurological and psychiatric disorders.

## Methods and Materials

*Subjects*: Nine healthy subjects (5 male and 4 female, ages from 21 to 55 years old with μ ± σ = 29.4 ± 11.1) participated in the study. The Institutional Review Board at Michigan State University approved the study, and written informed consent was obtained from all subjects prior to the study. All methods were performed in accordance with the institution’s relevant guidelines and regulations.

### Task paradigm

The task paradigm consists of a total of 24 task trials with 3 different tasks. Each trial comprises a 6-s task period followed by a 24-s rest period. Task 1 is a WR paradigm: an English word appears on the screen for 1.2 s and subjects read the word silently once. A total of five words are presented during the 6-s task period. Task 2 is a PV paradigm consisting of a total of 10 visual stimulation cycles during the 6-s task period. Each cycle consists of a 120 ms visual stimulus followed by a 480 ms blank screen^23^. The visual stimulus is a black-and-white striped pattern with a spatial frequency of 2.8 cycles per degree, and the blank screen with a small black square fixation mark at the center has the same mean luminance of the striped pattern. The size of visual field is 23 × 30 degree visual angle. Task 3 is a visually cured FT paradigm: the small black square fixation mark at the blank screen center changes to a small red cross during the 6-s task period to cue subjects to tap their right-hand five fingers. They were asked to tap fingers as quick as possible in a random order. The presentation of the three tasks is interleaved. During the 24-s rest period, subjects focus their eyes on the small black square fixation mark at the center of the blank screen and try not to think of anything.

### Image acquisition

Functional brain images were acquired on a GE 3.0 T clinical scanner with an 8-channel head coil using a gradient echo Echo-Planar-Imaging pulse sequence (TE/TR = 28/2500 ms, flip angle 80°, FOV 224 mm, matrix 64 × 64, slice thickness 3.5 mm, and spacing 0.0 mm). Thirty eight axial slices to cover the whole brain were scanned, and the first three volume images were discarded. The visual stimuli were projected onto a vertical screen placed inside the magnet bore using a MR-compatible Hyperion digital projection system with a 23 × 30 degree of visual angle placed at the back of the magnet room, the stimulation presentation was controlled by a PC equipped with E-Prime, and a BrainLogic Fiber Optic Button Response System with a pair of 5-button MR-compatible keypads was used to record subjects’ finger-tappings (Psychology Software Tools, Inc., Pittsburgh, PA). The participants viewed the screen through a mirror mounted on top of the head coil. For the participants who needed vision correction, MR-compatible lenses were used. Head movement was minimized by restraint using tape and cushions. Each participant first had a 12-min resting-state (rs) fMRI scan and then a 12-min task fMRI scan. Each scan yielded a total of 288 volume images (total time points N = 288). For the rs-fMRI scan, the participants were instructed to close their eyes and try not to think of anything but remain awake during the whole scan. Prior to the rs-fMRI scan, T2-weighted brain MR images with the same slices of the functional scans were acquired using a 2D T2-weighted pulse sequence. After the task-fMRI scan, T1-weighted whole-brain MR images were also acquired using a 3D IR-SPGR pulse sequence.

### Image preprocessing

Image preprocessing of the functional images was performed using AFNI (http://afni.nimh.nih.gov/afni), including (1) removing spikes from the signal intensity time course with the “3dDespike” routine; (2) slice-timing correction of the image acquisition time difference from slice to slice with the “3dTshift” routine; (3) motion correction of the images to align all volume images from the two functional scans to the very first volume image of 38 slices of the very first functional scan with the “3dvolreg” routine; (4) spatial filtering each volume image with a full-width-half-maximum (FWHM) of 0 (no filtering), 0.875, 1.75, 3.5, 4.0, 4.5, 5.25, and 6.0 mm, respectively, with the “3dmerge” routine; (5) computing the mean volume image of 38 slices for each FWHM value with the “3dTstat” routine; (6) bandpassing the signal intensity time course to the range of 0.009 Hz – 0.08 Hz for each FWHM value with the “3dBandpass” routine; and (7) computing the relative signal change (%) of the bandpassed signal intensity time course for each FWHM value with the “3dcalc” routine. After these preprocessing steps, further image analysis was carried out using in-house developed Matlab-based software algorithms.

### FAUPA determination

A statistical model and Matlab-based software algorithms have been developed and tested to determine FAUPA^13^. The determination consists of two major procedures. (1) For a given voxel, the voxel’s signal time course is used to compute *R*_*i*_ for each of the twenty-six surrounding voxels. If four or more voxels having *R*_*i*_ > 0.9, the mean time course of the 4 voxels with the four largest *R*_*i*_ is first computed and then used to compute *R*_*i*_ for each of these 4 voxels. Their μ and σ are calculated and used to establish a *R*_*i*_ threshold TH1 = μ − 1.645σ (one-tail probability < 5%). This mean time course is then used to compute *R*_*i*_ for all voxels within a cubic box that has 11 × 11 × 11 voxels with the given voxel at the center, and then these voxels are thresholded with TH1. The largest cluster with all voxels connected to each other and each voxel’s *R*_*i*_ > TH1 forms a region-of-interest (ROI). The ROI-mean time course is then computed and used to compute *R*_*i*_ for all voxels. Then, the μ and σ of the ROI are calculated and used to calculate a new TH1. After that, all voxels are thresholded with this new TH1, and this results in a new ROI. The new ROI is compared to the old ROI, and then this process is iterated until a stable ROI, i.e., the new and old are the same ROI, is reached or stops if the total number of voxels the ROI contains is larger than 29 or the iteration number is larger than 20. (The cutoff voxel number of 29 was chosen based on our preliminary analysis of the total number of voxels a FAUPA may contain.) (2) When a stable ROI is found, a second threshold TH2 = μ − 2.327σ (one-tail probability < 1%) is computed to establish a 4% statistical criterion, i.e., there is a 4% chance for a voxel’s *R*_*i*_ to be in the range TH2 < *R*_*i*_ < TH1. The total number (K) of voxels bordering the ROI is computed and used to calculate a criterion K_c_ = 4% × K. Then, the total number (L) of voxels bordering the ROI with *R*_*i*_ value in the range TH2 < *R*_*i*_ < TH1 is computed. If L ≤ K_c_, the stable ROI satisfies the statistical criterion and is identified as a FAUPA; otherwise, the ROI is discarded.

### Data availability

Both the original and processed fMRI images plus final research data related to this publication will be available to share upon request with a legitimate reason such as to validate the reported findings or to conduct a new analysis.
